# Effect of Nutraceutical Supplementation and Mediterranean Hypocaloric Diet on Calculated Steatosis Indices and Inflammation: Clinical and In Vitro Evidences

**DOI:** 10.1002/mnfr.70207

**Published:** 2025-09-06

**Authors:** Mariana Di Lorenzo, Luca Pipicelli, Laura Aurino, Concetta Sozio, Anna Palmiero, Domenico Palmieri, Marta Lombardi, Bruna Guida, Mariarosaria Santillo, Maria Serena Lonardo, Giuliana La Rosa, Simona Damiano

**Affiliations:** ^1^ Department of Clinical Medicine and Surgery Physiology Unit University of Naples “Federico II” Napoli Italy

**Keywords:** inflammation, leucoselect phytosome, NAFLD, nutraceutical supplementation, oxidative stress

## Abstract

This study evaluated the effect of a nutraceutical supplementation (NS) and Mediterranean hypocaloric diet (MHD) on hepatic steatosis indices (HSIs), γ‐glutamyl transferase (γGT), and lipid profile in adults with hyperlipidemia and nonalcoholic fatty liver disease (NAFLD). In vitro study on HepG2 cells explored potential molecular mechanisms. A retrospective study was conducted on 45 overweight/obese subjects (19 M) prescribed MHD with/without NS. Anthropometric data, biochemical parameters, HSIs, and γGT were collected at baseline and after 3 months. In vitro, cells were pretreated with single and mixed NS components and then with tumor necrosis factor α (TNFα) or fatty acids (FAs). Antioxidant and antiinflammatory activities were evaluated by fluorescence assays and quantitative polymerase chain reaction or enzyme‐linked immunosorbent assay; antiapoptotic effects by Western blot. After 3 months, all subjects improved anthropometric and biochemical parameters but only the combined MHD and NS treatment significantly reduced insulin resistance, HSIs, low‐density lipoprotein cholesterol, and γGT. In vitro, treatment with mixed NS components decreased TNFα‐/FAs‐induced reactive oxygen species. Combined treatment also modulated the inflammatory response by lowering interleukin‐6 and interleukin‐1β, increasing interleukin‐10 and pro‐caspase 8 expression. These findings suggest that NS, due to its antiinflammatory properties, represents a promising strategy for NAFLD management.

## Graphical abstract

This retrospective study evaluated the effects of nutraceutical supplementation (NS) and a Mediterranean hypocaloric diet (MHD) in NAFLD patients. Forty‐five overweight/obese subjects followed MHD with or without NS. After 3 months, only the MHD + NS group showed significant improvements in liver steatosis indices and inflammation. The in vitro study on HepG2 cells confirmed NS's antiinflammatory effects by reducing TNFα‐induced ROS, IL‐6, IL‐1β, and apoptosis while increasing IL‐10. Furthermore, NS is able to reduce ROS and IL‐6 induced by FAs in a steatotic model of HepG2 cells. These results support NS as a nutritional strategy for improving liver health in NAFLD patients. The figure was created with BioRender.com.

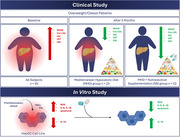

## 1 Introduction

In recent decades, overweight and obesity have frighteningly reached epidemic proportions and represent a significant source of global health concern [1, 2] as these conditions are associated with elevated risk of noncommunicable diseases (NCDs) [3] including hypertension [4, 5], Type 2 diabetes (T2D), dyslipidemia [6], nonalcoholic fatty liver disease (NAFLD) [7], and cardiovascular disease [5]. Rather than body mass index (BMI), recent evidence is shining the spotlight on the key role of dysfunctional adipose tissue that usually occurs due to pathological expansion of fat mass (FM) and/or unhealthy distribution of body fat [8] and serves as a gateway for the onset of these complications [6, 9, 10, 11]. Overall, it has been shown that in metabolically unhealthy obesity, which has dysfunctional adipose tissue, the storage capacity of the subcutaneous adipose tissue (SAT), the largest depot of white adipose tissue, is limited and chronic overeating leads to the accumulation of hypertrophic adipocytes at the visceral level and in ectopic organs (e.g., liver, skeletal muscle, and heart). This phenomenon is commonly referred to as “lipotoxicity” [9] and promotes inflammation and oxidative stress [12]. The hypertrophic adipocytes, indeed, undergo hypoxia and fibrosis with a concomitant increase in the secretion of proinflammatory cytokines such as monocyte chemoattractant protein‐1 (MCP‐1) tumor necrosis factor α (TNFα), interleukin‐6 (IL‐6), and interleukin 1β (IL‐1β) which, in addition to acting locally by amplifying inflammation on the adipose tissue itself, are released into circulation, eliciting a low‐grade chronic inflammation (LGCI). This LGCI is an important source of oxidative stress [13] and activates a vicious circle and acts on various organs and tissues promoting insulin resistance (IR) development [6, 12]. Oxidative stress occurs when there is an imbalance between small chemical species that contain one or more unpaired electrons that can oxidize other compounds named reactive oxygen species (ROS) formation and the activity of antioxidant defense systems, which play a key role in maintaining redox balance [14]. Visceral adipose tissue, IR, LGCI, and oxidative stress are widely recognized as the underlying mechanism linking components of the metabolic syndrome (MetS) and might play a major role also on NAFLD occurrence [15, 16, 17]. NAFLD, detected in the presence of histologic evidence of hepatic steatosis alone, is gradually increasing worldwide and represents the major chronic liver disease, as well as being acknowledged as the hepatic component of MetS [18]. Early diagnosis of NAFLD is crucial not only to prevent extrahepatic complications of the disease such as cardiovascular disease [19] but also to prevent progression to advanced stages of the disease as approximately one third of NAFLD may evolve into nonalcoholic steatohepatitis (NASH), which in turn may progress to cirrhosis and eventually to hepatocarcinoma [20]. Currently, no specific drugs are approved for NAFLD treatment. Therapeutic strategies to slow down or halt the progression of NAFLD consist mainly of lifestyle changes (diet, smoke/alcohol cessation, and physical activity) with the aim of reducing body weight and visceral adiposity, improving insulin sensitivity, and reducing cardiometabolic risk factors [21, 22]. Of interest, it has been shown that diet rich in antiinflammatory and antioxidants phytochemicals can be useful in treating NAFLD [23, 24]. In this perspective, a novel nutraceutical supplementation (NS) with Leucoselect Phytosome (LSP), red yeast rice, monacolin K (MK), polycosanols, and folic acid (Ostacol Plus, Agaton) has been formulated for dyslipidemia treatment in people at low cardiovascular risk [25]. As a matter of fact, natural phytochemicals such as octacosanol (OCT) could be used as supplement for the prevention and treatment of obesity‐related metabolic diseases thus representing a valuable tool for treating the pathophysiological changes that accompany and worsen the metabolic picture of NAFLD [26, 27]. OCT is the main component of a long‐chain saturated fatty alcohol mix called policosanol, which is purified from natural sources such as wheat, rice bran, sugarcane, and beeswax [28]. It has been shown that polycosanols are able to reduce cholesterol levels, prevent lipoprotein peroxidation, and thus reduce the development of atherosclerosis in a variety of experimental models [29]. Specifically, it is well documented that OCT plays an important role in the prevention of obesity, metabolic, and cardiovascular disorders [26], through the regulation of 3‐hydroxy‐3‐methylglutaryl coenzyme A (HMG‐CoA) reductase activity, which is responsible for the sterol biosynthesis [30]. In recent years, more attention has been focused on the beneficial effects of some nutraceuticals as silymarin, astaxanthin, resveratrol, quercetin, curcumin, and β/α‐glucans biologically active compounds commonly used to improve health status, in the treatment of numerous diseases, including NAFLD [31, 32, 33]. In this regard, it has been demonstrated that NS (vitamin E, l‐glutathione, silymarin, and hepato‐active compounds) in synergy with the Mediterranean diet could exert protective effect on liver in individuals with NAFLD [16]. Another molecule gaining attention is MK, the natural form of lovastatin, it is a metabolite isolated from *Monascus* spp. (red yeast) and is a powerful inhibitor of HMG‐CoA reductase and its consumption has lipid lowering action [34]. An interesting in vitro study using HepG2 cells demonstrated that MK reduces the lipid content through Sirtuin 1/AMP‐activated protein kinase (SIRT1/AMPK) pathway, promoting catabolism and inhibiting lipid anabolism [35]. Additionally, an experimental study on NAFLD mice model demonstrated that chronic administration of MK is able to reduce insulin levels, liver fat deposits, and obesity‐related parameters thereby reducing NAFLD progression [34, 36]. In agreement with these animal data, an observational study showed that MK treatment in subjects with NAFLD significantly reduced of the homeostatic model assessment (HOMA) index serum glutamic‐pyruvic transaminase (SGPT) supporting the hypothesis of a beneficial effect of this molecule on both insulin sensitivity and inflammatory response [17]. In addition, nutraceutical research is focusing on polyphenols and flavonoids for their antioxidant power and numerous beneficial effects on the nervous [33], cardiovascular [37], and enteric [38] systems. Among these molecules, Leucoselect (LS), a proanthocyanidin extracted from the seeds of *Vitis vinifera*, the common grapevine, is known for its antioxidant activity and its role in improving microcirculation [39]. LSP, a special formulation which combines LS with soy lecithin, has been widely used as a supplement for cardiovascular health. Specifically, it improves the total antioxidant capacity of plasma and reduces LDL cholesterol susceptibility. Moreover, it can increase serum levels of eicosapentaenoic acid (EPA), docosahexaenoic acid (DHA), ω‐3 polyunsaturated fatty acids (n‐3 PUFAs), and prostaglandins (PGs) E3, which has antiinflammatory properties [40]; further studies use this special formulation as a hypolipidemic nutraceutical in dyslipidemic patients [25].

Thus, considering this nutraceutical formulation, the purpose of the present study was to evaluate its effects in combination with Mediterranean diet on hepatic indices as well as on lipid profile and nutrition status in overweight/obese adults living with hyperlipidemia and NAFLD. Furthermore, to better clarify the biomolecular mechanisms by which this NS could exert a hepatoprotective action, an in vitro study was conducted using human hepatocarcinoma cell line (HepG2) to assess its antioxidant and antiinflammatory properties.

## 2 Materials and Methods

### 2.1 Clinical Study

#### 2.1.1 Subjects’ Selection and Study Design

This observational monocentric retrospective study was carried out from January 2021 to December 2023 at the Outpatient Clinic of “Diet therapy in transplantation and chronic renal failure”, “Federico II” University of Naples. The study protocol was approved by the Ethics Committee of our Institution on May 28, 2024 (Project identification code 152/2024). Informed consent was obtained from all subjects involved in the study. The eligibility criteria were as follows:

- Subjects with a diagnosis of NAFLD obtained by ultrasound medical report;
- Age between 18 and 65 years old;
- BMI ≥ 25 and ≤ 40 kg/m^2^;
- Plasma levels of total cholesterol (Col‐TOT) 220–270 mg/dL;
- Plasma levels of low‐density lipoprotein cholesterol (Col‐LDL) 130–160 mg/dL.

On the other hands, the exclusion criteria included: pregnancy, breastfeeding, alcohol consumption (≥30 g per day for men and ≥20 g per day for women) [41], diabetes, cancer, depression or neurological disorders, autoimmune disease, chronic kidney disease, pancreatic and liver diseases, familial hypercholesterolemia, smoke, cholesterol‐lowering therapy, or any other condition considered to be inappropriate for the study. A total of 45 individuals were retrospectively selected and prescribed a Mediterranean hypocaloric diet (MHD) and 1 capsule/daily of NS for 3 months. After data collection, it was found that only 22 subjects had followed both MHD and nutraceutical prescription. Therefore, the subjects were divided into two groups: MHD group (control group) and MHD + NS group. The chemical composition of the NS capsule is summarized in Table 1. Demographic and clinical characteristics, biochemical parameters, pharmacological treatments, anthropometric measurements, body composition as well as hepatic steatosis indices (HSIs) [16, 18] and data about physical activity [42] were recorded at baseline and after 3 months.

**TABLE 1:** Chemical composition of a capsule of nutraceutical compound.

Nutritional Information ingredients	Amount per serving
Leucoselect Phytosome	300 mg
Red yeast rice extract	99 mg
Monacolin K	2,97 mg
Polycosanols	10 mg
Octacosanol	9 mg
Folic acid	200 mcg

*Note*: This nutraceutical is registered in the “Register of supplements” of the Ministry of Health (code 110454).

#### 2.1.2 Anthropometric, Clinical, and Biochemical Parameters

##### 2.1.2.1 Anthropometric Parameters and Body Composition

Nutritional status was investigated by anthropometric measurements and body composition. To ensure accuracy, measurements were taken by a trained clinical nutritionist. Subjects were instructed to wear minimal and/or snug‐fitting clothing. Body height (m) and weight (kg) were detected using a calibrated balance beam scale and a stadiometer (Seca 711; Seca Hamburg, Germany), and BMI was briefly calculated. Waist circumference (WC) in cm was measured using a flexible nonelastic measuring tape. The recommended location for WC measurement was in the midpoint between the lower rib and the top of the iliac crest [43]. In addition, waist‐to‐height ratio (WHtR) was calculated as the WC (cm) divided by the height (cm) [44]. To evaluate the body composition, bioelectrical impedance analysis (BIA) with a single‐frequency measurement (50 kHz) was conducted using the Akern device (BIA 101 BIVA PRO AKERN srl, Florence, Italy) [45], and the exam was performed according to ESPEN guidelines [46]. Prior to testing, subjects were instructed to maintain hydration and avoid vigorous exercise for 24 h. They were also advised to refrain from alcohol and caffeine consumption for 12 h before the test. Subjects were asked to remove any metal objects. They were positioned in a supine position on a flat surface, with their limbs extended and not touching each other. Electrodes were placed on the right hand and foot (for the measurement to be accurate and reproducible, the electrodes were attached to specific locations with a distance ≥5 cm from each other). In addition, estimates of the fat‐free mass (FFM), FM, total body water (TBW), expressed in %, and phase angle (*Φ*) were collected. Furthermore, Food Frequency Questionnaires (FFQs) were performed to assess the eating habits of the subjects.

##### 2.1.2.2 Clinical and Biochemical Parameters

Blood pressure was measured using an aneroid sphygmomanometer by medical doctor. Subjects were instructed to avoid food, caffeine, smoking, and vigorous exercise for at least 30 min before measurement, and they were also asked to sit quietly for 5 min. They were seated in a comfortable chair with their back support and feet flat on the floor. The arm employed for measurement was positioned at heart level. The cuff was placed around the upper arm, approximately 1–2 cm above the elbow, ensuring that it was snug but not too tight. Next, the stethoscope was placed over the brachial artery, and the cuff was inflated to 20–30 mmHg above the expected systolic pressure. The cuff was then gradually deflated at a rate of approximately 2–3 mmHg per second. The first Korotkoff sound was noted as systolic blood pressure, and the point at which the sounds disappeared was recorded as the diastolic blood pressure. Three measurements with 2 min intervals were performed [47, 48]. Overnight fasting (12 h) venous blood samples data of subjects were collected for the registration of levels of glucose (Gly), Col‐TOT, Col‐LDL, high‐density lipoprotein cholesterol (Col‐HDL), triglycerides (TGs), SGPT, and serum glutamic‐oxaloacetic transaminase (SGOT). In addition, TGs/Col‐HDL ratio was derived to assess IR [42]. Lastly, to define potential oxidative stress and inflammation status of subjects, serum gamma‐glutamyl transferase (γGT) was also collected [49, 50].

#### 2.1.3 Hepatic Indices

The following hepatic indices were calculated:

- Hepatic steatosis index (HSI): 8 × (SGPT/SGOT ratio) + BMI (+2, if women; +2, if diabetes mellitus) [51];
- Visceral adiposity index (VAI) [52]:
  ∘ VAI (men): (WC)/[39.68 + (1.88 × BMI)] × (TGs/1.03) × (1.31/Col‐HDL);
  ∘ VAI (women): (WC)/[36.58 + (1.89 × BMI)] × (TGs/0.81) × (1.52/Col‐HDL).

#### 2.1.4 Diet Prescription and Assessment of Dietary Compliance

All subjects received a detailed dietary hypocaloric diet based on the principles of the Mediterranean diet, with a calorie restriction of 40% of the total energy needs. The macronutrient distribution was established as follows: carbohydrates 55%–60%, lipids 20%–25%, and proteins 10%–15% of total daily caloric intake [16]. Dietary compliance (data not shown) and nutraceutical consumption were assessed at baseline and at the end of the study. FFQs were performed to assess the adherence to diet, and diary records were asked to the subjects to record the time of consumption of the nutraceutical.

### 2.2 In Vitro Study

#### 2.2.1 Chemicals

MK (Sigma–Aldrich, 75330‐75‐5), LSP, polycosanols with 90% OCT, and folic acid (FA) were purchased from Agaton s.r.l. With regard to red yeast rice standardized to 3% MK, we tested only the active compound, MK. To obtain the solution of individual compounds each was dissolved in the respective solvent while respecting the solubility of the substance. MK was prepared in DMSO; LSP solution was prepared in phosphate buffered saline (PBS) which contained 130.9 mM NaCl, 5.1 mM Na_2_HPO_4_, and 1.5 mM KH_2_PO_4_ at pH 7.4; OCT was dissolved in ethanol (EtOH) at 70°C for 10 min, and then a solution was prepared to be added to the cells in EtOH–PBS at a ratio of 1:4; FAs solution was prepared in NaOH 1 mM.

#### 2.2.2 Cell Culture

HepG2 is a cell line exhibiting epithelial‐like morphology that was derived from hepatocellular carcinoma. This cell line was cultured in high‐glucose Dulbecco's Modified Eagle's Medium (DMEM), containing 4.5 g/L glucose (GIBCO, Thermo Fisher Scientific, Waltham, MA, USA), supplemented with 10% fetal bovine serum (FBS; GIBCO, Thermo Fisher Scientific), 1% l‐glutamine (GIBCO, Thermo Fisher Scientific), and 100 U/mL penicillin and 100 µg/mL streptomycin (complete DMEM). Cells were cultured at 37°C in a 5% (v/v) CO_2_ incubator.

#### 2.2.3 In Vitro Treatment (Nutraceutical Mixes)

The cells were treated with different nutraceutical compounds combination, either taken individually or within a mix that mimics the proportions of the nutraceutical (Ostacol Plus, Agaton s.r.l., Italy), as described in specific experiments. Two mixes were obtained (MIX1 and MIX2) prepared to respect the proportions of the components of the nutraceutical, as described in Table 2.

**TABLE 2:** Percentual and concentrations of components of the nutraceutical mixes.

	%	MIX1	MIX2
Leucoselect Phytosome	71	10 µg/mL	20 µg/mL
Polycosanols with 90% octacosanol	2.2	310 ng/mL	620 ng/mL
Monacolin K	0.74	100 ng/mL	200 ng/mL
Folic acid	0.05	7.1 ng/mL	14.2 ng/mL

#### 2.2.4 Fluorometric Determination of ROS

ROS levels were determined using membrane‐permeant ROS‐sensitive fluorogenic probe 5,6‐carboxy‐2‐,7‐dichlorofluoresceindiacetate (DCFH‐DA) (Molecular Probes, Thermo Fisher Scientific). DCFH‐DA is a probe that freely crosses the plasma membrane and is hydrolyzed in the cytosol to form the carboxylate anion DCFH. The oxidation of DCFH leads to the formation of fluorescent DCF (reported Ex/Em: 485–500 nm/515–530 nm). HepG2 cells grew to semiconfluence in 24 multiwell plates in complete DMEM and subsequently were starved for 24 h with 0.2% FBS medium and where necessary, cells were treated with the two different concentrations of MIX or the individual components. As a positive control to assess the increase in ROS levels, cells were preincubated for 4 h with 10 ng/mL TNFα (300‐01, PrepoTech, Thermo Fisher Scientific), or in presence of fatty acids (FAs) 1 mM. FAs were obtained in a 2:1 ratio of oleic acid (OA, Sigma–Aldrich O7501) and palmitic acid (PA, Sigma–Aldrich P9767). The cells were washed twice with PBS and incubated at 37°C in DMEM without FBS in the presence of 10 µM DCFH‐DA for 10 min and shaking each 5 min, then the cells were washed three times with PBS containing 10 mM glucose, 1.2 mM MgCl_2_, and 1.2 mM CaCl_2_ and dichlorofluorescein (DCF). Fluorescence was measured with the Fluoroskan Ascent FL plate reader at room temperature under shaking conditions (Thermo Electron Oy, Vantaa, Finland), and the data were analyzed with Ascent software version 2.6.

#### 2.2.5 DHE (Dihydroethidium) Analysis

Intracellular superoxide levels were assessed using the fluorescent probe dihydroxyethidium bromide (DHE) 10 µM (Molecular Probes, Thermo Fisher Scientific), a cell‐permeable fluorescent probe that reacts with superoxide anions to form 2‐hydroxyethidium, a red fluorescent product (excitation/emission 518/605 nm). The experimental procedure, including cell preparation, staining, and fixation, was performed according to the protocol described by La Rosa et al. [53], where full methodological details are available. Briefly, HepG2 cells were cultured on coverslips in 12‐well plates and starved in the presence or absence of MIX2, followed by stimulation with TNFα 10 nM for 4 h and processed for DHE staining. Cells were analyzed using a Leica DMi8 fluorescence microscope, and image quantification was performed with ImageJ software (version 1.8) by calculating total corrected cellular fluorescence (TCCF), according to the protocol used by McCloy et al. [54], from 50 cells per condition in three independent experiments in triplicate.

#### 2.2.6 Western Blotting Analysis

Western blot analysis was performed as previously described by La Rosa et al. [53], where full methodological details are provided. In brief, HepG2 cells were grown to semiconfluence in 6‐well plates and starved for 24 h with or without MIX2 and in the absence or presence of TNFα 10 ng/mL. Protein lysates were extracted using RIPA buffer supplemented with phosphatase and protease inhibitors. Equal amounts of protein (30 µg) were separated by 10% SDS‐PAGE and transferred onto nitrocellulose membranes (GE Healthcare, Amersham PI, UK). Membranes were blocked with 5% BSA in TBS‐T for 1 h at room temperature, then incubated overnight with an anti‐pro‐caspase 8 antibody (4790, Cell Signaling Technology, Danvers, MA, USA), followed by a peroxidase‐conjugated anti‐rabbit secondary antibody (Blotting Grade Goat Anti‐Rabbit IgG H + L Horseradish Peroxidase Conjugate, Bio‐Rad, Hercules, CA, USA). Signal detection was performed using an enhanced chemiluminescence (ECL) system (Immobilon Western Chemiluminescent HRP Substrate WBKLS010055, Merck Millipore, Darmstadt, Germany) and visualized with a ChemiDoc XRS+ imaging system (Bio‐Rad). Membranes were stripped and reprobed with an anti‐GAPDH antibody (E‐AB‐40337, Elabscience, Houston, TX, USA) for normalization. Band intensities were quantified by densitometry using ImageJ software (version 1.8, National Institutes of Health, Bethesda, MD, USA).

#### 2.2.7 Gene Expression Analysis

Total RNA extraction, reverse transcription, and qPCR were performed as previously described by Menale et al. [55], where full methodological details are provided. HepG2 cells were grown to semiconfluence in 12‐well plates and starved for 24 h with or without MIX2, then treated with TNFα 10 ng/mL for 1 or 6 h. Total RNA was extracted using PureZOL Reagent (Bio‐Rad) following the manufacturer's instructions. Reverse transcription was carried out using 1 µg total RNA and All‐In‐One 5X RT MasterMix with gDNA Removal (ABM, New York, NY, USA). The qPCR was performed using iTaq Universal SYBR Green Supermix (Bio‐Rad) on a CFX Connect Real‐Time PCR Detection System (Bio‐Rad) with the following cycling conditions: 95°C for 3 min, then 40 cycles of 95°C for 15 s, and 60°C for 30 s, followed by melting curve analysis. Relative gene expression was calculated using the 2^−ΔΔCt^ method normalized to 18s housekeeping control gene. The normalized expression was calculated as a fold change mRNA level versus control condition. Primer sequences are listed in Table 3.

**TABLE 3:** The forward and reverse primer of the genes (*IL‐6*, *IL‐10*, *IL‐1β*, *18s*).

Gene	Forward primer 5′ → 3′	Reverse primer 3′ → 5′
*IL‐6*	TGTCTTCCTCACCGATTCCT	ACCACCCGAGCTCTGTCTTACTC
*Il‐10*	GTGAAAACAAGAGCAAGGCCG	TCGCCACCCTGATGTCTCA
*IL‐1β*	GTCTTCAACAAGATAGAAGTCAAG	TGTCTTCCTCACCGATTGCT
*18s*	GCGCTACACTGACTGGCTC	CATCCAATCGGTAGTAGCGAC

#### 2.2.8 Sandwich‐ELISA Assay

HepG2 cells were grown to semiconfluence in 12 multiwell plates in complete DMEM and subsequently were starved for 24 h with medium containing 0.2% FBS. Where necessary, cells were treated with MIX2 and in the absence or presence of FAs (1 mM, OA:PA, 2:1). After 24 h, supernatants were collected. A Sandwich‐ELISA assay for the detection of IL‐6 was performed using a commercial kit, following the manufacturer's instructions (Human IL‐6 ELISA Kit, E‐EL‐H6156, Elabscience, Wuhan, CN). Optical densities (ODs) at 450 nm were measured using iMark Microplate Absorbance Reader (Bio‐Rad), and concentrations were determined by interpolation from the standard curve using nonlinear regression (four‐parameter logistic, 4PL) in GraphPad Prism 9 (GraphPad Software, San Diego, CA, USA).

#### 2.2.9 Oil Red‐O Staining

For Oil Red‐O staining (Sigma–Aldrich O0625), HepG2 cells were grown to semiconfluence in 12 multiwell plates in complete DMEM and subsequently were starved for 24 h with medium containing 0.2% FBS. Where necessary, cells were treated in absence or presence of FAs (1 mM, OA:PA, 2:1). Cells were then fixed with 4% PFA for 15 min at room temperature and washed with PBS. A 0.18% Oil Red O solution prepared in 60% isopropanol and 40% water was used. Images were acquired using a Leica DMi1 microscope with a 20× objective. Quantification was performed by dissolving the retained dye in 99% isopropanol, and absorbance was measured at 490 nm using iMark Microplate Absorbance Reader (Bio‐Rad).

#### 2.2.10 BODIPY 493/503 Analysis

For the analysis of lipid accumulation, HepG2 cells were grown to semiconfluence on coverslip in 12‐well plates in complete DMEM and subsequently starved for 24 h in medium containing 0.2% FBS. Then, cells were then treated in the absence or presence of FAs (1 mM, OA:PA, 2:1). Lipid droplets (LDs) were detected using the BODIPY 493/503 probe (Sigma–Aldrich, St. Louis, MO, USA) at a concentration of 1 µg/mL for 30 min (excitation/emission: 493/504 nm). Nuclei were stained with DAPI (Sigma–Aldrich). Images were acquired using a Leica DMi8 fluorescence microscope equipped with Leica Application Suite LAS X imaging software. BODIPY‐positive LD pixel areas were quantified using ImageJ software (National Institutes of Health). Five images per sample were analyzed.

#### 2.2.11 Statistical Analysis

For clinical study, continuous variables are expressed as mean (M) ± standard deviation (SD). Categorical variables are presented as number and percentages. Paired samples *t* test, independent samples *t* test, and chi‐squared test were performed, respectively. A *p* value < 0.05 was considered statistically significant. Statistical analysis was performed with IBM Corp. Released 2023. IBM SPSS Statistics for Windows, Version 29.0.2.0 Armonk, NY: IBM Corp.

Data about in vitro study are presented as mean ± SEM. As appropriately indicated, differences between groups were compared using ANOVA followed by Bonferroni's post hoc test to correct for multiple comparisons. A *p* value < 0.05 was considered statistically significant. In addition, statistical differences between two groups were assessed using Student's *t* test for unpaired samples, and differences were considered statistically significant at *p* < 0.05.

## 3 Results

### 3.1 Clinical Study Results

**FIGURE 1:**
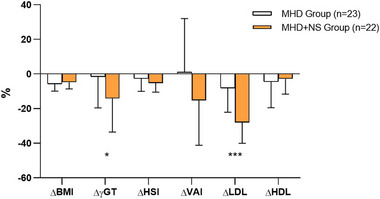
Comparison of ΔBMI, ΔγGT, ΔHSI, ΔVAI, ΔLDL, and ΔHDL between MHD and MHD + NS group. Data are expressed as mean (M) ± standard deviation (SD). The exact sample size was *n* = 23 for the MHD group and *n* = 22 for the MHD + NS group. Δ was obtained from biological replicates. Statistical analysis was performed using independent sample *t* test. ***p < 0.001; *p < 0.05 versus MHD group. γGT, gamma‐glutamyl transferase; BMI, body mass index; Col‐HDL, high‐density lipoprotein–cholesterol; Col‐LDL, low‐density lipoprotein–cholesterol; HSI, hepatic steatosis index; MHD, Mediterranean hypocaloric diet; NS, nutraceutical supplementation; VAI, visceral adiposity index.

A total of 45 ambulatory individuals with NAFLD and elevated blood levels of both total and LDL cholesterol were included. Of the overall population, 22 (49%) reported taking NS compared to 23 (51%) who had refused this treatment, and all of them followed the Mediterranean diet prescription. Of the total subject population studied, the mean age was 52.4 ± 11.9 years, 42.2% were men and the mean BMI was 30.6 kg/m^2^. Physical inactivity was found in 100% of subjects. No significant differences for all analyzed variables between MHD group and MHD + NS group emerged at baseline (*p* = ns). Table 4 summarizes the baseline explored features of the two groups. The mean Col‐TOT and Col‐LDL levels in MHD group were similar to the mean levels in the MHD + NS group (231.3 ± 12 mg/dL vs. 235 ± 15.7 mg/dL, respectively; and 153 ± 16.7 mg/dL vs. 158 ± 15.1 mg/dL, respectively; *p* = ns). Similarly, no difference was found in transaminases baseline levels (Table 4). Likewise, the noninvasive hepatic derivable indices, HSI, VAI, were similar between MHD + NS group and MHD group (*p* = ns) (Table 4). In particular, the baseline HSI analysis revealed that an HSI score > 36 was reported in both groups. In addition, serum γ‐glutamyltransferase (γGT) baseline levels were compared, and no significant differences between MHD group and MHD + NS group have emerged (22.9 ± 13.5 U/L vs. 27.4 ± 15.7 U/L, respectively; *p* = ns) (Table 4). Other biochemical parameters were described in Table 4. Comparison of baseline and 3 months data of both groups is shown in Table 5. First, posttreatment decrease in BW, BMI. and FM was observed in both groups when compared to the baseline data (*p* < 0.01). After 3 months, both groups showed significant improvements in Col‐TOT, Col‐LDL, TGs, and WHtR (*p* < 0.05). Further analysis revealed a significant decrease in glucose levels with concomitant reduction in TGs/Col‐HDL ratio, γGT, HSI, and VAI only in MHD + NS group compared to baseline data (*p* < 0.05), while stable Col‐HDL levels were observed after 3 months of treatment (*p* = ns) (Table 5). Remarkably, the MHD + NS group exhibited a significant percentage decrease of LDL and γGT compared to MHD group data (−28% vs. −8.1% and −14.1% vs. −1.5%; respectively; *p* < 0.05) (see Figure 1).

**TABLE 4:** Baseline characteristics of study population.

	All subjects	MHD group	MHD + NS group	
	*n* = 45	*n* = 23	*n* = 22	*p* value
Age (years) (M ± SD)	52.4 ± 11.9	53.7 ± 9.9	51 ± 14	*ns*
Male, *n* (%)	19 (42.22%)	9 (20%)	10 (22.22%)	*ns*
BW (kg) (M ± SD)	83.1 ± 13	83.5 ± 12.2	82.8 ± 14.2	*ns*
BMI (kg/m^2^) (M ± SD)	30.6 ± 4.1	31.2 ± 3.9	30 ± 4.3	*ns*
WC (cm) (M ± SD)	98.6 ± 9	98.9 ± 8.6	98.3 ± 9.8	*ns*
WHtR (M ± SD)	0.6 ± 0	0.6 ± 0	0.5 ± 0	*ns*
TBW (%) (M ± SD)	49.9 ± 5.9	49.8 ± 6.1	50.1 ± 5.9	*ns*
FFM (%) (M ± SD)	65.6 ± 8.9	65.2 ± 9.1	67 ± 7.9	*ns*
FM (%) (M ± SD)	33.5 ± 8.1	33.7 ± 8.5	32.9 ± 7.9	*ns*
Glucose (mg/dL) (M ± SD)	94.7 ± 15.2	98.3 ± 21.8	89 ± 10.6	*ns*
Col‐TOT (mg/dL) (M ± SD)	233.1 ± 13.7	231.3 ± 12	235 ± 15.7	*ns*
Col‐LDL (mg/dL) (M ± SD)	155.6 ± 15.8	153 ± 16.7	158 ± 15.1	*ns*
Col‐HDL (mg/dL) (M ± SD)	57.3 ± 14.2	59 ± 14	55.5 ± 14.9	*ns*
TG (mg/dL) (M ± SD)	113.5 ± 44.8	113.2 ± 52.5	113.7 ± 38	*ns*
TG/HDL ratio (M ± SD)	2.1 ± 1.2	2 ± 1.3	2.2 ± 1.1	*ns*
GOT (U/L) (M ± SD)	22.1 ± 4.2	20.7 ± 4.1	23.3 ± 4.2	*ns*
GPT (U/L) (M ± SD)	23.4 ± 8.7	22.1 ± 9.8	25.1 ± 7.9	*ns*
γGT (U/L) (M ± SD)	24.5 ± 13.9	22.9 ± 13.5	27.4 ± 15.7	*ns*
HSI (M ± SD)	40.3 ± 6.1	41 ± 6.2	40.3 ± 6.8	*ns*
VAI (M ± SD)	3.4 ± 1.9	3.2 ± 2	3.6 ± 1.8	*ns*
SBP (mmHg) (M ± SD)	129.2 ± 14.7	131.1 ± 16.2	126.8 ± 13.2	*ns*
DBP (mmHg) (M ± SD)	84 ± 9.3	86.1 ± 10.9	82.7 ± 8.3	*ns*

*Note*: Continuous variables are expressed as mean ± standard deviation (SD). Categorical variable is indicated as number/percentage (%).

Abbreviations: γGT, gamma‐glutamyl transferase; BMI, body mass index; BW, body weight; Col‐HDL, high‐density lipoprotein–cholesterol; Col‐LDL, low‐density lipoprotein–cholesterol; Col‐TOT, total cholesterol; DBP, diastolic blood pressure; FFM, fat‐free mass; FM, fat mass; GOT, glutamic‐oxalacetic transaminase; GPT, glutamyl pyruvic transaminase; HSI, hepatic steatosis index; SBP, systolic blood pressure; TBW, total body water; TG, triglyceride; VAI, visceral adiposity index; WC, waist circumference; WHtR, waist to height ratio.

**TABLE 5:** Anthropometric measurements, body composition, clinical and biochemical parameters, hepatic and adiposity indices of MHD and MHD + NS groups at baseline and after 3 months of treatment.

	MHD group			MHD + NS group		
	*n* = 23			*n* = 22		
	Baseline	After 3 months	P1	Baseline	After 3 months	P2
BW (kg) (M ± SD)	83.5 ± 12.2	79 ± 13	<0.001	82.8 ± 14.2	79.2 ± 12.7	<0.001
BMI (kg/m^2^) (M ± SD)	31.2 ± 3.9	29.4 ± 4	<0.001	30 ± 4.3	28.5 ± 3.9	<0.001
WC (cm) (M ± SD)	98.9 ± 8.6	95.8 ± 9.7	<0.001	98.3 ± 9.8	94.4 ± 7.8	<0.05
WHtR (M ± SD)	0.6 ± 0	0.5 ± 0	<0.001	0.5 ± 0	0.5 ± 0	<0.05
TBW (%) (M ± SD)	49.8 ± 6.1	51.2 ± 6.4	<0.05	50.1 ± 5.9	51.7 ± 7.7	*ns*
FFM (%) (M ± SD)	65.2 ± 9.1	68.6 ± 8.5	*ns*	67 ± 7.9	69.3 ± 9	<0.001
FM (%) (M ± SD)	33.7 ± 8.5	31.3 ± 8.5	<0.01	32.9 ± 7.9	30.2 ± 8.5	<0.001
Glucose (mg/dL) (M ± SD)	98.3 ± 21.8	95.1 ± 16.1	*ns*	89 ± 10.6	82.7 ± 7.1	<0.05
Col‐TOT (mg/dL) (M ± SD)	231.3 ± 12	212.8 ± 19.7	<0.001	235 ± 15.7	182.2 ± 26.5	<0.001
Col‐LDL (mg/dL) (M ± SD)	153 ± 16.7	140 ± 21.6	<0.05	158 ± 15.1	113.3 ± 19	<0.001
Col‐HDL (mg/dL) (M ± SD)	59 ± 14	55.1 ± 10.5	0.05	55.5 ± 14.9	53.6 ± 14.3	*ns*
TG (mg/dL) (M ± SD)	113.2 ± 52.5	97.5 ± 34.7	<0.05	113.7 ± 38	89.7 ± 30.3	<0.01
TG/HDL ratio (M ± SD)	2 ± 1.3	1.8 ± 0.9	*ns*	2.2 ± 1.1	1.8 ± 0.8	<0.01
GOT (U/L) (M ± SD)	20.7 ± 4.1	19.8 ± 5.9	*ns*	23.3 ± 4.2	21.2 ± 5.7	*ns*
GPT (U/L) (M ± SD)	22.1 ± 9.8	22.3 ± 10.9	*ns*	25.1 ± 7.9	22.7 ± 8.5	*ns*
γGT (U/L) (M ± SD)	22.9 ± 13.5	22.1 ± 13.2	*ns*	27.4 ± 15.7	21.3 ± 8	<0.05
HSI (M ± SD)	41 ± 6.2	40 ± 6.9	*ns*	40.3 ± 6.8	38 ± 5.8	<0.01
VAI (M ± SD)	3.2 ± 2	2.8 ± 1	*ns*	3.6 ± 1.8	2.9 ± 1.2	<0.01
SBP (mmHg) (M ± SD)	131.1 ± 16.2	127.9 ± 10.3	*ns*	126.8 ± 13.2	123.9 ± 15.5	*ns*
DBP (mmHg) (M ± SD)	86.1 ± 10.9	83.5 ± 7	*ns*	82.7 ± 8.3	77.8 ± 9.9	<0.05

*Note*: Continuous variables are expressed as mean  ±  standard deviation (SD). P1 (*p* value 1), baseline MHD group versus MHD group after 3 months; P2 (*p* value 2), baseline MHD + NS group versus MHD + NS group after 3 months.

Abbreviations: γGT, gamma‐glutamyl transferase; BMI, body mass index; BW, body weight; Col‐LDL, low‐density lipoprotein–cholesterol; Col‐HDL, high‐density lipoprotein–cholesterol; Col‐TOT, total cholesterol; DBP, diastolic blood pressure; FFM, fat‐free mass; FM, fat mass; GOT, glutamic‐oxalacetic transaminase; GPT, glutamyl pyruvic transaminase; HSI, hepatic steatosis index; SBP, systolic blood pressure; TBW, total body water; TG, triglyceride; VAI, visceral adiposity index; WC, waist circumference; WHtR, waist height ratio.

### 3.2 In Vitro Study

#### 3.2.1 Antioxidant Effect of Individual and Mixed Components of NS in HepG2 Cell Line

**FIGURE 2:**
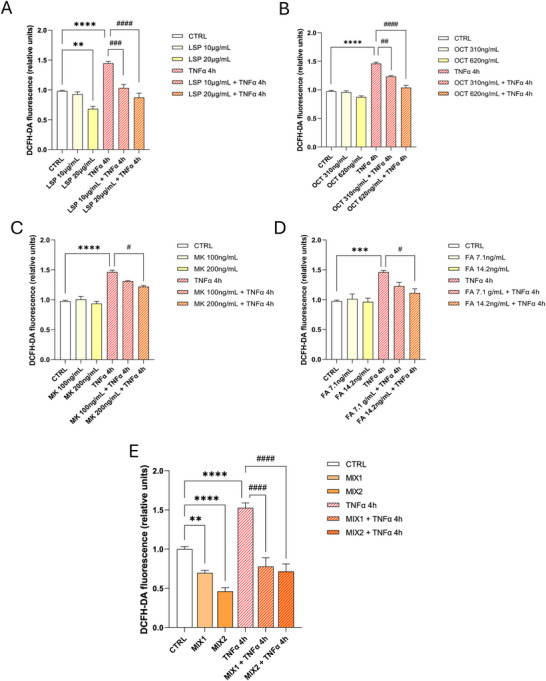
Individual compounds and nutraceutical mixes reduce the TNFα‐induced ROS production. (A–E) Intracellular ROS levels measured by DCFH‐DA fluorescence assay. HepG2 cells were preincubated with the individual compounds at varying concentrations or with the two nutraceutical mixes (MIX1 and MIX2) for 24 h. Subsequently, cells were stimulated with 10 ng/mL TNFα for 4 h before the incubation with 10 µM of the ROS‐sensitive probe DCHF‐DA. ROS levels were measured by fluorometric analysis. The graphs show the mean ± SEM values from three independent experiments in triplicate. Statistical analysis was performed using the ordinary one‐way ANOVA with Bonferroni's post hoc test correction. **p < 0.005 versus CTRL; ***p < 0.001 versus CTRL; ****p < 0.0001 versus CTRL; #*p* <0.05 versus TNFα; ##*p* < 0.005 versus TNFα; ###*p* <0.001 versus TNFα; ####*p* < 0.0001 versus TNFα. ANOVA, analysis of variance; CTRL, control; DCFH‐DA, 5,6‐carboxy‐2‐,7‐dichlorofluoresceindiacetate; ROS, reactive oxygen species; TNFα, tumor necrosis factor α.

**FIGURE 3:**
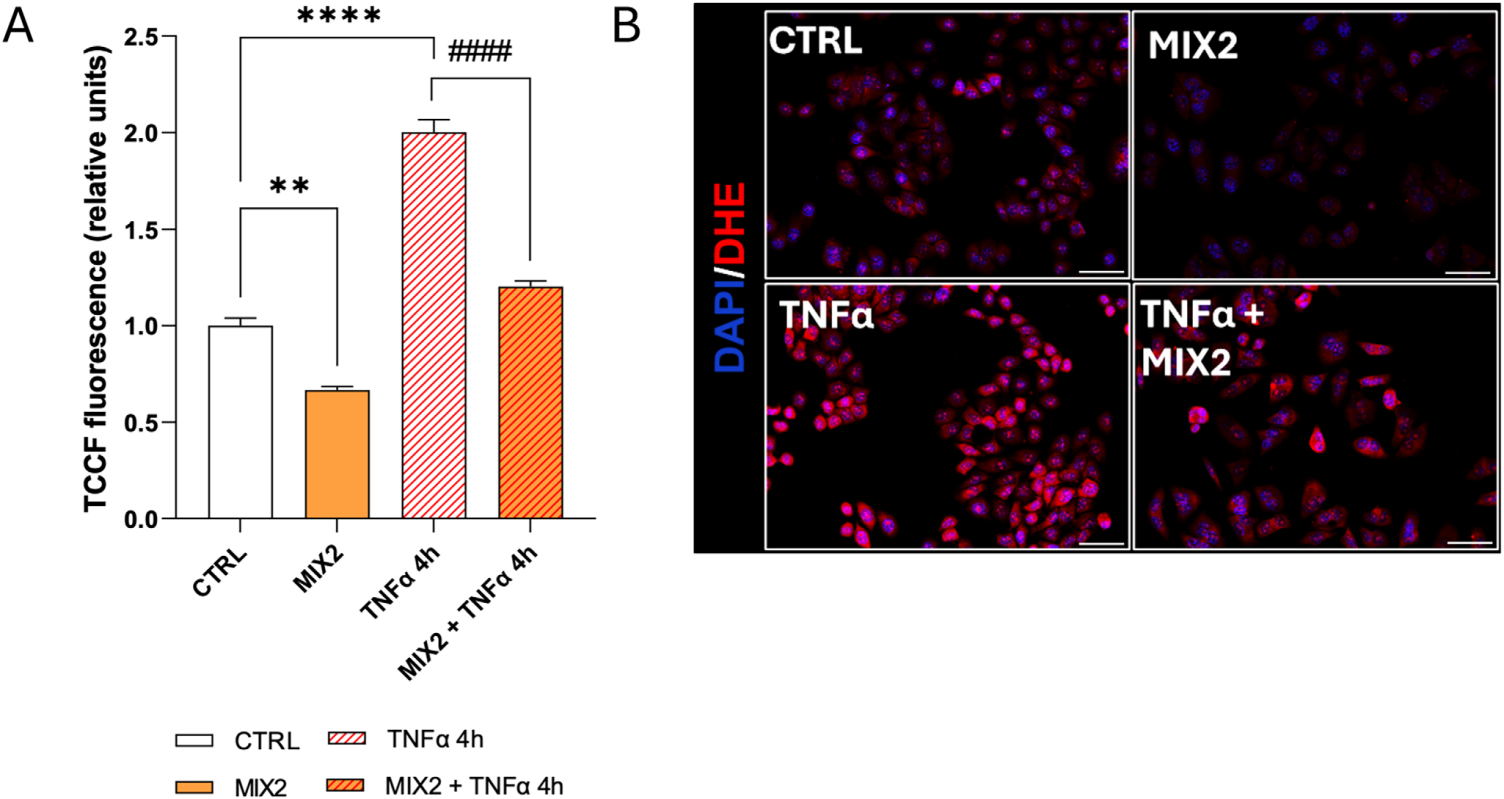
MIX2 reduces intracellular superoxide levels. HepG2 cells were starved for 24 h in medium containing 0.2% FBS in the absence (CTRL) or presence of MIX 2 and then with TNFα 10 ng/mL for 4 h. (A) The histogram shows total corrected cellular fluorescence (TCCF) values, obtained from quantitative analysis of fluorescence microscopy images (scale bar 50 µm) of DHE (10 µM) stained cells. For each sample, 50 cells were analyzed. Values are mean ± SEM from three independent experiments performed in triplicate. (B) Representative fluorescence microscopy images of HepG2 cells stained with DHE. Statistical analysis was performed using the ordinary one‐way ANOVA with Bonferroni's post hoc test correction. **p <0.005 versus CTRL; ****p < 0.0001 versus CTRL; ####*p* < 0.0001 versus TNFα. ANOVA, analysis of variance; CTRL, control; DHE, dihydroxyethidium bromide; FBS, fetal bovine serum; TNFα, tumor necrosis factor α.

To complement clinical data and to elucidate the molecular mechanisms underlying the antiinflammatory effect of NS, in vitro experiments were conducted on human hepatocarcinoma cells (HepG2), commonly employed as a cellular model in drug metabolism and hepatotoxicity studies [56, 57]. To evaluate the independent effects of the components of the NS (Ostacol Plus), we tested each compound individually on basal and TNFα‐induced ROS production in HepG2 cells. Two concentrations of each component were chosen for the experiments, both of which demonstrated no cytotoxic effects (data not shown). ROS and cytokines are major mediators of the inflammatory process [58]. Cytokines like TNFα can generate ROS as secondary messengers, amplifying inflammatory responses. Using the DCFH‐DA fluorescence assay, we observed that individual components did not significantly reduce basal ROS levels, except for LSP at higher concentrations (20 µg/mL). In contrast, all individual components showed an inhibitory effect on TNFα‐induced ROS levels. Statistically significant reductions in ROS levels relative to TNFα were observed with LSP (10 and 20 µg/mL) and OCT (310 and 620 ng/mL), while FA and MK exhibited effects only at their highest concentrations (14.2 and 200 ng/mL, respectively) (Figure 2A–D). After evaluating the effects of individual components on ROS levels, we reproduced two mixes (MIX1 and MIX2) both matching the composition of NS (LSP 71%; OCT 2.2%; MK 0.74%; and FA 0.05%) but differing in the concentrations of individual components (Table 2) to examine their effects on HepG2 cells. Viability assays confirmed that these mixes were noncytotoxic (data not shown). The antioxidant effects of MIX1 and MIX2 on HepG2 cells were evaluated using the DCFH‐DA fluorescence assay. Both mixes significantly reduced basal intracellular ROS levels compared to the control (CTRL). Notably, when tested individually, none of the components significantly affected basal ROS levels, except for LSP. However, the reduction observed with the complete mix was greater than that induced by LSP alone, indicating that while LSP plays a primary role in the ROS‐lowering effect, the overall efficacy is further improved in the context of the full formulation. To demonstrate the antioxidant effects of MIX1 and MIX2 under inflammatory condition, we measured intracellular ROS levels in TNFα‐treated HepG2 cells (4 h) in the absence or presence of the mixes. The histogram shows a significant increase in intracellular ROS levels in TNFα‐stimulated cells and their reduction in the presence of MIX1 and MIX2 (Figure 2E). The antioxidant effect of MIX2 was stronger than MIX1, prompting us to focus further analyses on MIX2. DCFH‐DA assay data were confirmed with DHE, a fluorescent probe specific for intracellular superoxide showing a statistically significant reduction in cells treated with MIX2 compared to CTRL. The histogram also shows an increase in intracellular superoxide levels when cells were treated with TNFα and a reduction in the presence of MIX2 (Figure 3A,B).

#### 3.2.2 The Nutraceutical Mixture Attenuates Inflammation and Apoptosis in TNFα‐Stimulated HepG2 Cells

**FIGURE 4:**
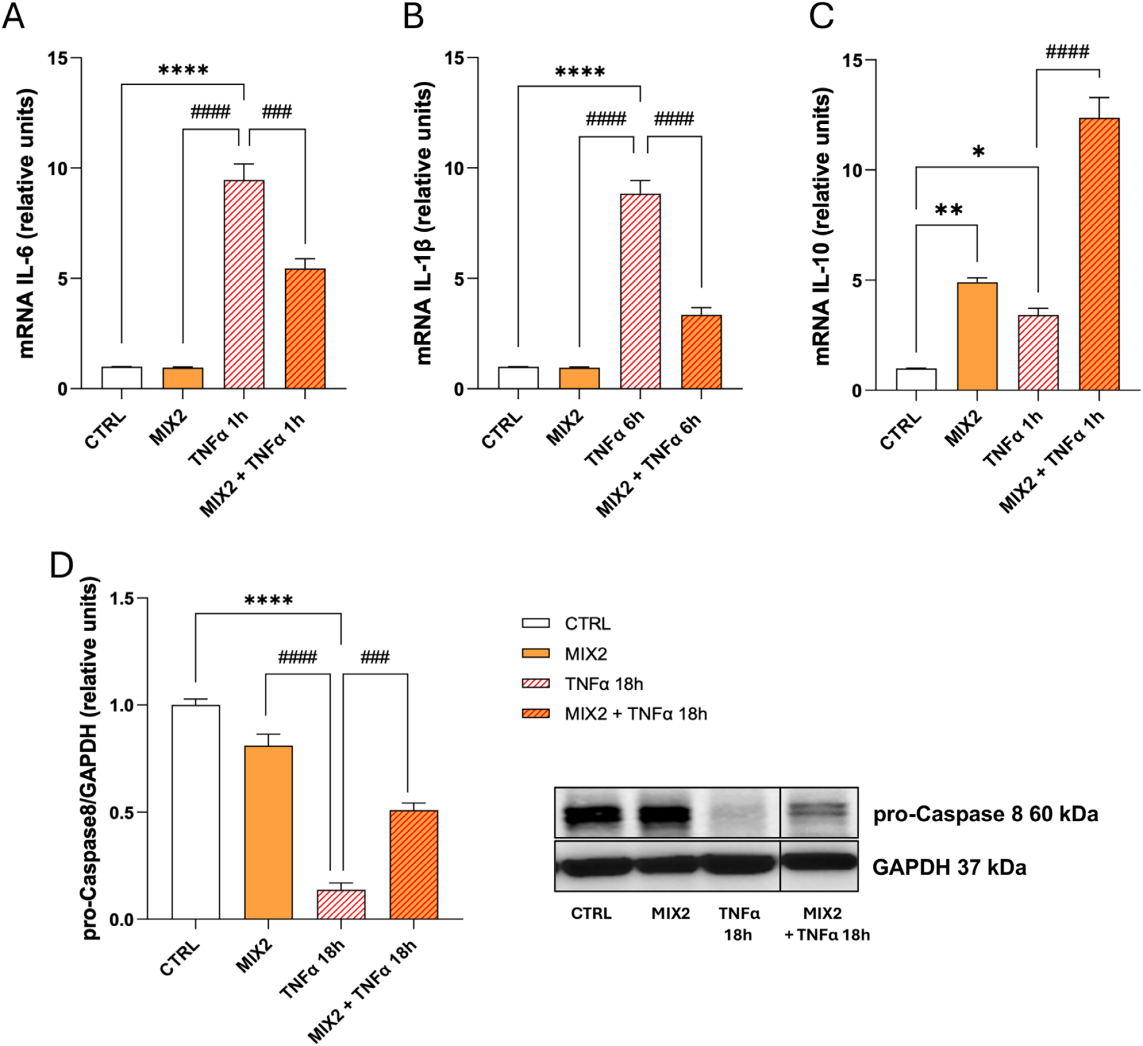
MIX2 blocks the proinflammatory and proapoptotic pathways activated by TNFα. HepG2 cells were starved for 24 h in medium containing 0.2% FBS in the absence (CTRL) or presence of MIX2 and then with TNFα 10 ng/mL for the indicated times. (A–C) qPCR analysis was performed to measure mRNA levels of (A) *IL‐6*, (B) *IL‐1β*, and (C) *IL‐10*. The histograms show the values normalized to *18s* rRNA (means ± SEM) relative to the CTRL of three independent experiments in triplicate. (D) Western blot analysis of pro‐caspase 8 protein levels. The histogram shows the densitometric values (means ± SEM) relative to the CTRL, of pro‐caspase 8 bands normalized to GAPDH of three independent experiments in triplicate. The omitted lanes do not contain data relevant to the conclusions of the study. The corresponding source data are available in the Supporting Information file “Source Data for Figure 4”. Statistical analysis was performed using the ordinary one‐way ANOVA with Bonferroni's post hoc test correction. *p < 0.05 versus CTRL; **p < 0.005 versus CTRL; ****p < 0.0001 versus CTRL; ###*p* < 0.001 versus TNFα; ####*p* < 0.0001 versus TNFα. ANOVA, analysis of variance; CTRL, control; FBS, fetal bovine serum; TNFα, tumor necrosis factor α.

In agreement with the results obtained from clinical studies, suggesting an antiinflammatory action of the nutraceutical, we evaluated the antiinflammatory effect of MIX2 on the TNFα‐treated HepG2 cells. It is known that these cells can express, albeit at low levels, pro‐ and antiinflammatory cytokines [59, 60, 61, 62]. We therefore evaluated the gene expression levels of the principal cytokines released during inflammatory processes, specifically the antiinflammatory cytokine IL‐10 and the proinflammatory cytokines IL‐6 and IL‐1β. It is well known in the literature that the increase of these cytokines during inflammation occurs at different time points; for this reason, we chose 1 and 6 h for the treatment with TNFα [63, 64, 65]. In cells treated with an inflammatory stimulus, MIX2 significantly reduced the gene expression of IL‐6 and IL‐1β compared to TNFα alone (Figure 4A,B). Additionally, MIX2 treatment enhanced IL‐10 expression levels, even beyond the increase observed as an adaptive response to TNFα stimulation [66]. This enhancement highlights the antiinflammatory potential of MIX2 (Figure 4C). Inflammatory processes can activate apoptotic pathways specifically through activation of caspases, the main effector enzymes of apoptosis. Thus, in the presence of apoptotic processes, there is a reduction in the native inactive form of caspase 8, pro‐caspase 8 [53]. As shown in Figure 4D, TNFα (18 h) decreases protein expression levels of pro‐caspase 8 compared to CTRL. Conversely, MIX2 treatment in the presence of TNFα significantly increases the levels of pro‐caspase 8, compared to TNFα alone. These data suggest that the activation of the extrinsic apoptotic pathway by this cytokine is inhibited by MIX2, highlighting its dual antiinflammatory and antiapoptotic effects supporting its therapeutic potential in counteracting oxidative stress and inflammation‐induced damage.

#### 3.2.3 Antiinflammatory Effects of a Nutraceutical Mixture in a Cellular Model of Hepatic Steatosis

**FIGURE 5:**
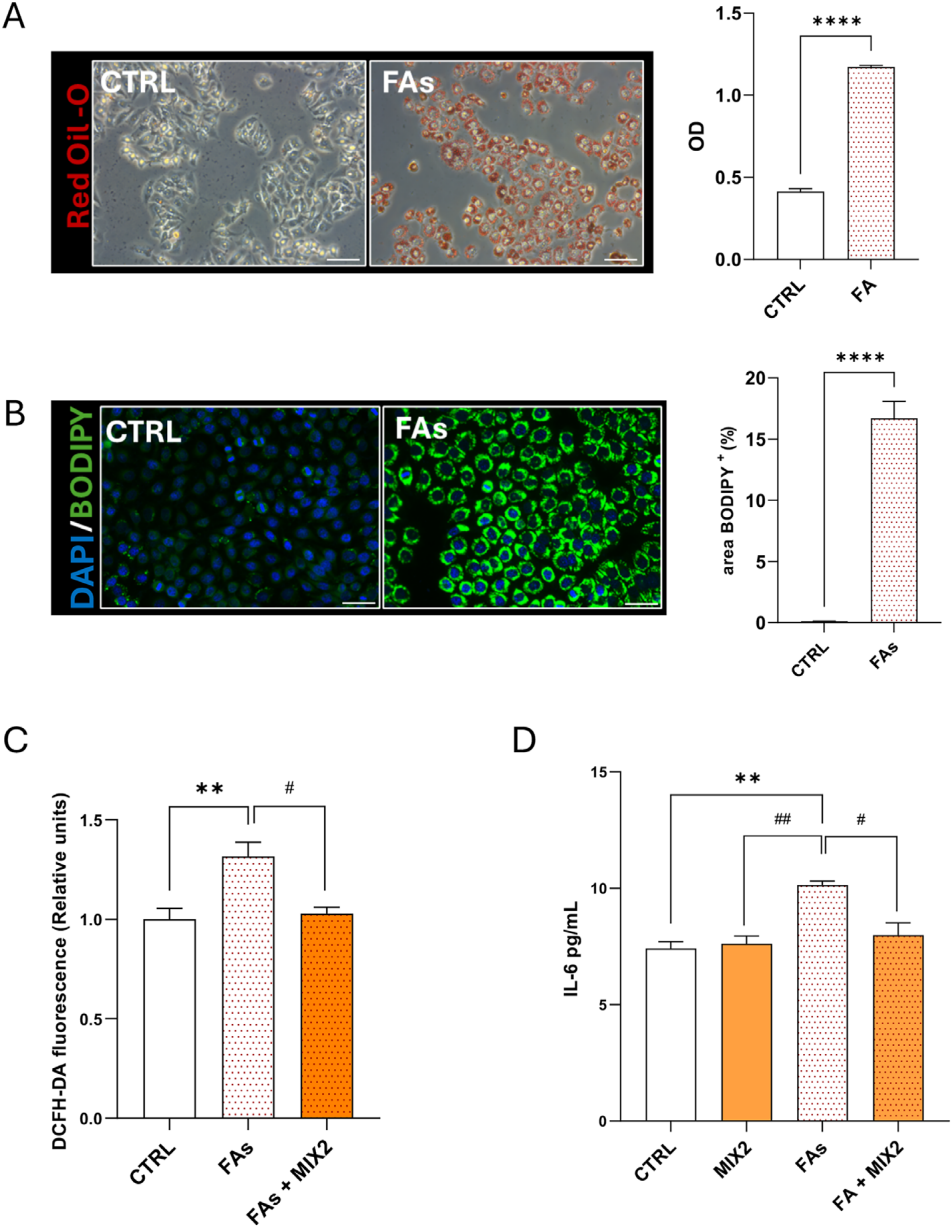
MIX2 attenuates FAs‐induced oxidative and inflammatory responses. For the assessment of lipid accumulation, HepG2 cells were starved for 24 h in medium containing 0.2% FBS in the absence (CTRL) or in presence of FAs. (A) Representative images for Oil Red O and corresponding OD quantification. Scale bar: 50 µm. (B) BODIPY 493/503 fluorescence staining and relative quantification expressed as LDs^+^ area (percentage of total pixels). Scale bar: 50 µm. (C) Intracellular ROS levels were measured using 10 µM of DCFH‐DA in HepG2 cells treated with or without FAs and/or MIX2. The histogram shows the ROS levels quantified by fluorometric analysis. (D) The detection of IL‐6 in the conditioned medium was measured using a sandwich‐ELISA assay. Concentrations were determined by interpolation using nonlinear regression (four‐parameter logistic, 4PL) based on the standard curve. All the histograms show the mean ± SEM values from three independent experiments in triplicate. Statistical analysis was performed using Student's *t* test or the Ordinary one‐way ANOVA with Bonferroni's post hoc test correction for the multiple comparisons. **p < 0.005 versus CTRL; ****p < 0.0001 versus CTRL; #*p* < 0.05 versus FAs; ####*p* < 0.0001 versus FAs. ANOVA, analysis of variance; CTRL, control; FA, fatty acid; DCFH‐DA, 5,6‐carboxy‐2‐,7‐dichlorofluoresceindiacetate; FBS, fetal bovine serum; LD, lipid droplet; OD, optical density; ROS, reactive oxygen species.

A cellular model of hepatic steatosis was established by treating HepG2 cells with a mixture of FAs, specifically OA and PA, at a 2:1 ratio [67] and a final concentration of 1 mM. The use of this OA:PA ratio is supported by evidence showing that OA and PA are the predominant FAs present in hepatic TGs of both healthy and NAFLD subjects [68]. Therefore, this in vitro model is widely used to study NAFLD, as it replicates key features of the disease, such as intracellular lipid accumulation and activation of inflammatory pathways, under low‐toxicity conditions, thereby allowing the investigation of early mechanisms involved in increased susceptibility to cellular damage [69]. Lipid accumulation was assessed using Oil Red O staining and BODIPY 493/503 fluorescent probe, confirming the development of steatosis after 24 h of FAs treatment, as shown in Figure 5A,B. Although cotreatment with the MIX2 did not reduce lipid accumulation after 24 h (data not shown), MIX2 exhibited marked antioxidant and antiinflammatory activity. Indeed, FAs treatment significantly increased ROS production, as determined by DCFH‐DA fluorescence and the cotreatment with MIX2 significantly reduced ROS levels, restoring them to values comparable to CTRL (Figure 5C). A similar effect was observed for the proinflammatory cytokine IL‐6, measured in the cell culture supernatant by Sandwich‐ELISA assay. Although FAs treatment markedly increased IL‐6 secretion, cotreatment with MIX2 attenuated this response, bringing cytokine levels close to baseline (Figure 5D). These findings support the antiinflammatory potential of MIX2 and its ability to counteract the proinflammatory effects induced by FAs in HepG2 cells.

## 4 Discussion

Obesity, the main hallmark of the MetS, modifies the phenotype of white adipose tissue, characterized by the presence of dysfunctional and inflamed adipocytes that secrete, both locally and systemically, proinflammatory cytokines such as TNFα, IL‐1β, and IL‐6, which alter the functionality of other target organs [70] including the liver [71]. Recent evidence has already turned light on the potential role of several classes of bioactive compounds in managing and ameliorating complications of MetS [72, 73]. Currently, no specific drugs are approved for NAFLD treatment. Results obtained in this study indicate that overweight/obese individuals suffering from hyperlipidemia and NAFLD treated with MHD + NS showed significant improvements in HSIs and γGT biomarker, plasma lipid profile, as well as insulin sensitivity. Our clinical study showed that a significant reduction in both total and LDL cholesterol in both MHD either MHD + NS groups. This is conceivable considering that the Mediterranean diet is widely recognized for its ability to reduce the risk factors of inflammatory disorders like MetS and to improve liver fat content and insulin sensitivity in patients with NAFLD [74, 75, 76]. However, in agreement with a previous study [25], the observed lipid‐lowering effects were stronger in MHD + NS group, indicating that the two treatments together have a greater beneficial effects. In detail, the absence of a control group treated with NS alone (without MHD) did not allow us to clarify whether the observed effects in anthropometric and biochemical parameters related to NAFLD in the NS + MHD group were attributable to the combined action of both treatments or whether NS alone was sufficient to determine these results. Specifically, the study design inherently reflects the standard approach and current clinical practice, in which dietary supplements such as NS are always considered an adjunct to, and not a substitute for, the lifestyle modifications that are the primary management strategies for NAFLD [76]. In addition, a group treated with NS alone was not included in the present study, as all subjects were overweight/obese, for whom dietary intervention is the first and essential step in clinical management.

Newsworthy, only subjects belonging to MHD + NS group showed a significant reduction in both VAI and HSI indices, supporting the additional protective effects of NS to the diet. This finding is of particular interest as the control of adiposity represents one of the important solutions to improve NAFLD [77]; previous studies have indeed reported that fat distribution and a larger area of visceral adipose tissue exert greater effect on NAFLD [78]. Similarly, only in the latter group it has also been observed a significant improvement in metabolic parameters such as glycemia and TGs/HDL‐Col ratio, indicating an improvement in insulin sensitivity. Improved insulin sensitivity plays a key role in the management of NAFLD as the dysfunction of the insulin pathway causes the flow of FAs to the liver, increases TG synthesis and storage, inflammation, steatosis, and oxidative stress in the body [79]. Oxidative stress plays a key role in inflammatory processes, participates in the T‐cell activation [80], and contributes to subsequent cellular damage; increased oxidative stress and systemic inflammation are believed to be involved in the pathogenesis of MetS and NAFLD. Our clinical data demonstrated the antiinflammatory and antioxidant role of this NS, as a significant decrease in γGT was observed. γGT is an independent marker of both systemic inflammation and oxidative stress [81], as it exerts a central role in the extracellular catabolism of the antioxidant glutathione [82] that facilitates ROS scavenging [83]. For these reasons, the present study is especially noteworthy. The in vitro analysis using the HepG2 further elucidated the molecular mechanisms underlying these clinical benefits, highlighting the ability of NS in modulating ROS levels involved in inflammation and apoptosis. Intracellular ROS can act as second messengers in the inflammatory response induced by several cytokines including TNFα [53]. Indeed, cells incubated with this cytokine showed an increase in intracellular ROS levels, which are reversed in the presence of both the individual components and mixes of NS. The redox imbalance activates downstream inflammatory and apoptotic pathways; indeed, we observed that in the presence of TNFα, there is an activation of caspase 8, the main enzyme of the extrinsic apoptosis pathway and that this effect is reversed in the presence of NS.

Interestingly, LSP at 10 µg/mL alone did not significantly reduce ROS levels compared to untreated cells, suggesting a limited intrinsic antioxidant activity at this concentration. However, when combined with TNFα, the same dose of LSP was able to significantly reduce ROS levels, indicating a context‐dependent antioxidant effect. In particular, the MIX1 formulation, which contains LSP at 10 µg/mL together with other bioactive compounds, exerted a reduction in ROS levels both in the presence and absence of TNFα, in contrast to LSP 10 µg/mL alone where the effect occurs only in the presence of TNFα. Similarly, MIX2, which includes a higher dose of LSP (20 µg/mL), showed an even more pronounced effect. These results suggest that the increased antioxidant capacity of the mixtures cannot be attributed solely to LSP, but rather to the cooperative action of the combined components. This highlights the potential benefit of using a multicomponent nutraceutical formulation to counteract oxidative stress in inflammatory conditions.

The ability of NS to modulate proinflammatory and antiinflammatory cytokine levels further supports its potential as a therapeutic agent. By reducing IL‐6 and IL‐1β expression while enhancing IL‐10 levels, NS demonstrates a dual effect: mitigating inflammatory damage and promoting an adaptive immune response. This cytokine modulation may help to combat inflammation and oxidative stress, which are central to NAFLD pathogenesis. Finally, the antiapoptotic effects of NS have significant implications for the pathogenesis of NAFLD. Chronic oxidative stress and inflammation contribute to NAFLD progression, promoting hepatocyte apoptosis through pathways such as caspase activation [84]. The ability of NS to increase pro‐caspase 8 levels indicates an inhibition of the extrinsic apoptotic pathway, which is critical for preserving hepatocyte integrity.

To place the observed effects within a disease‐relevant context, we employed a well‐established in vitro model of hepatic steatosis based on FAs exposure. Although lipid content remained relatively unchanged after 24 h of treatment, MIX2 exerted a clear antioxidant and antiinflammatory effect in this model. FAs‐induced ROS generation, as assessed by DCFH‐DA staining, was significantly reduced by cotreatment with MIX2, restoring ROS levels close to baseline. Similarly, MIX2 attenuated the secretion of the proinflammatory cytokine IL‐6 induced by FAs. These results are consistent with those previously obtained with TNFα stimulation and further support the antiinflammatory potential of MIX2, even in the presence of the metabolic stress associated with steatosis. This highlights the potential of the formulation to modulate stress‐related pathways involved in NAFLD pathogenesis, possibly preceding changes in hepatic fat accumulation. Overall, these in vitro results align with the observed clinical improvements and provide mechanistic evidence supporting the hepatic antiinflammatory role of this nutraceutical.

According to the obtained results, it can be said that the supplementation with new NS composed of LSP, red yeast rice, MK, polycosanols, and folic acid (Ostacol Plus, Agaton) can be effective in improving lipid profile, insulin resistance, and hepatic indices due to its antioxidant, antiinflammatory, and antiapoptotic effects. Therefore, it represents a valuable tool for the treatment and prevention of NAFLD.

## Ethics Statement

The study was conducted in accordance with the Decla‐ration of Helsinki and approved by the Ethics Committee of the “Federico II” University Medical School of Naples on May 28 2024 (Project identification code 152/2024).

## Consent

Informed consent was obtained from all subjects involved in the study.

## Conflicts of Interest

The authors declare no conflicts of interest.

## Supporting information




**Supporting file 1**: mnfr70207‐sup‐0001‐SuppMat.pdf

## Data Availability

The clinical data are stored in a database at the Department of Clinical Medicine and Surgery, Nutrition Physiology Unit, University “Federico II” of Naples, Naples 80131, Italy. It is available upon request to Bruna Guida, who is a co‐author of this paper.
