# Supplementary figures and images for: Effect of Nutraceutical Supplementation and Mediterranean Hypocaloric Diet on Calculated Steatosis Indices and Inflammation: Clinical and In Vitro Evidences
Source: Mol Nutr Food Res. 2025 Sep 6;69(21):e70207. doi: 10.1002/mnfr.70207 (PMC12581743; doi:10.1002/mnfr.70207)

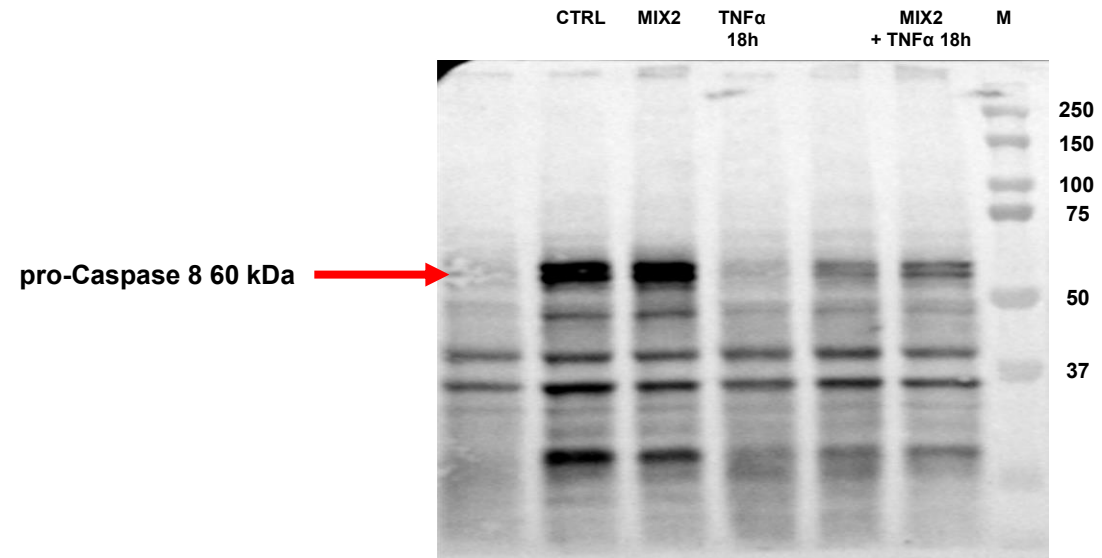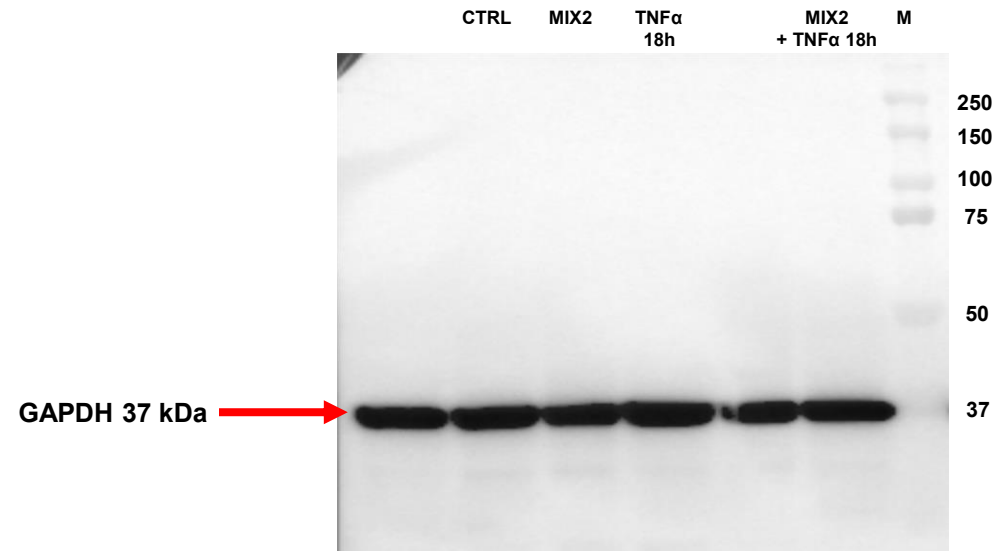

Supplement: Supplementary file 1 — Supporting file 1: mnfr70207‐sup‐0001‐SuppMat.pdf [file MNFR-69-e70207-s001.pdf]
